# Plant Autophagy: An Intricate Process Controlled by Various Signaling Pathways

**DOI:** 10.3389/fpls.2021.754982

**Published:** 2021-09-23

**Authors:** Pingping Wang, Tongtong Wang, Jingyi Han, Ming Li, Yanxiu Zhao, Tong Su, Changle Ma

**Affiliations:** Shandong Provincial Key Laboratory of Plant Stress, College of Life Sciences, Shandong Normal University, Jinan, China

**Keywords:** autophagy, signaling, nutrient, stress, plant hormone

## Abstract

Autophagy is a ubiquitous process used widely across plant cells to degrade cellular material and is an important regulator of plant growth and various environmental stress responses in plants. The initiation and dynamics of autophagy in plant cells are precisely controlled according to the developmental stage of the plant and changes in the environment, which are transduced into intracellular signaling pathways. These signaling pathways often regulate autophagy by mediating TOR (Target of Rapamycin) kinase activity, an important regulator of autophagy initiation; however, some also act via TOR-independent pathways. Under nutrient starvation, TOR activity is suppressed through glucose or ROS (reactive oxygen species) signaling, thereby promoting the initiation of autophagy. Under stresses, autophagy can be regulated by the regulatory networks connecting stresses, ROS and plant hormones, and in turn, autophagy regulates ROS levels and hormone signaling. This review focuses on the latest research progress in the mechanism of different external signals regulating autophagy.

## Introduction

As immobile organisms, plants are subjected to various environmental stresses, such as nutrient deficiency, heat, salt, drought, cold, and pathogen infection (Zhu, 2016). Because these stresses can seriously impact normal plant growth and physiology, plants have evolved a complex set of regulatory mechanisms allowing them to adapt to adverse conditions. One particularly important response mechanism is the degradation of damaged or superfluous proteins and organelle structures through autophagy to maintain homeostasis of intracellular substances and to recycle nutrients (Marshall and Vierstra, 2018). Under normal conditions, low levels of constitutive autophagy activity are important for plant growth and development. Additionally, autophagy can be significantly induced to help plants resist adverse stress conditions (Wang et al., 2018a). Therefore, regulation of the timing and levels of autophagy are vital for plant survival.

Autophagy was first described by Christian de Duve in 1963 (Klionsky, 2008). It was not until the 1990s that the first autophagy gene *Apg1* (*Autophagy 1*), later changed to *autophagy-related gene ATG1*, was identified in yeast (Matsuura et al., 1997). Since then, more than 30 ATGs have been identified in yeast (Wen and Klionsky, 2016). It is now thought that autophagy is present in all eukaryotes, sharing evolutionarily conserved core mechanisms (Liu and Bassham, 2012). Many homologs of yeast ATGs have been identified in plants (Hanaoka et al., 2002). In *Arabidopsis*, more than 40 ATGs have been found, most of which are homologs of yeast ATGs (Chung, 2019). Recently, great advances have been made in revealing the regulatory mechanisms of autophagy in plants and this review builds on recent research to present the latest understanding of the regulation of autophagy by various signaling pathways.

## The Mechanisms of Plant Autophagy

**FIGURE 1:**
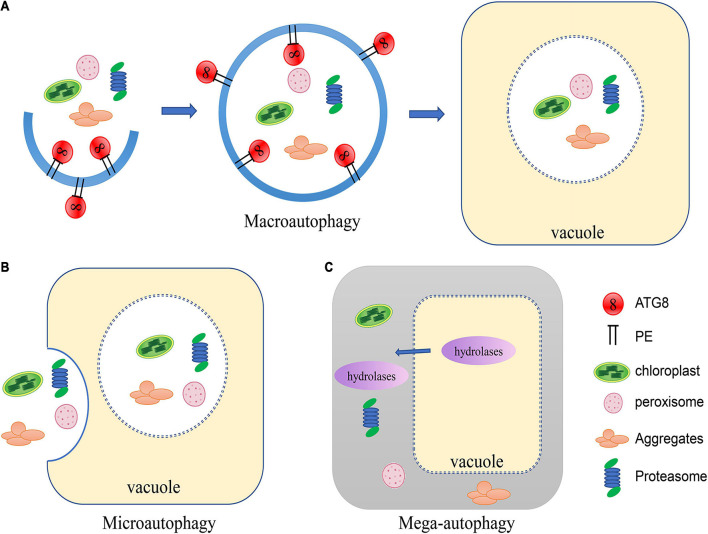
Schematic of the three types of autophagy in plants. **(A)** In macroautophagy, a cup-shaped phagophore is formed and closes into vesicular autophagosomes as it encircles cytosolic substances. The autophagosome is subsequently delivered to the vacuole. The outer membrane of the autophagosome fuses with the tonoplast and the internal vesicle is released to the vacuolar lumen for degradation. **(B)** In microautophagy, cytosolic materials are gathered on the tonoplast and directly captured by the vacuole through invagination to be degraded. **(C)** During mega-autophagy, the vacuolar membrane is permeabilized and ruptures, releasing large amounts of hydrolases into the cytoplasm, causing indiscriminate degradation of cytoplasmic materials.

Three forms of autophagy have been described in plants: microautophagy, macroautophagy and mega-autophagy (Marshall and Vierstra, 2018; Avin-Wittenberg, 2019; Gou et al., 2019; Figure 1). In microautophagy, cytosolic materials are gathered on the surface of the vacuole and directly captured through invagination of the tonoplast, forming membrane-wrapped autophagic bodies which will be degraded in the vacuole (Marshall and Vierstra, 2018). In contrast, macroautophagy is characterized by the formation of autophagosomes encapsulated in a bilayer membrane (Le Bars et al., 2014). A cup-shaped phagophore is formed at the phagophore assembly site (Zhuang et al., 2013). The membrane of the phagophore then elongates as it encircles cytosolic substances and closes off to form vesicular autophagosomes (Le Bars et al., 2014). The autophagosome is subsequently delivered to the vacuole (Ketelaar et al., 2004). The outer membrane of the autophagosome fuses with the tonoplast and the internal vesicle is released to the vacuolar lumen for degradation. Mega-autophagy is an extreme form of autophagy that so far has only been found in plants, and thus is not as well characterized as the main types of autophagy (Van Doorn and Papini, 2013). During mega-autophagy, the vacuolar membrane is permeabilized and ruptures, releasing large amounts of hydrolases into the cytoplasm, causing indiscriminate degradation of cytoplasmic materials (Hatsugai et al., 2004; Van Doorn and Papini, 2013). Mega-autophagy occurs in plants during developmental PCD (programmed cell death), which is a result of normal development as well as abiotic stresses, but does not occur in pathogen-induced PCD. Additionally, mega-autophagy leads to cell death rather than cell remobilization or stress resistance. Distinct from canonical autophagy, mega-autophagy does not involve capture of cellular components into the vacuole. No *ATG* genes have been reported to be involved in mega-autophagy. Therefore, mega-autophagy is often referred to as autolysis.

The underlying mechanism of macroautophagy (autophagy for short) has been more clearly characterized than that of microautophagy. A series of ATG proteins mediate the entire process of macroautophagy. The initiation of autophagy can be regulated by an essential serine/threonine kinase, TOR (Target of Rapamycin), which negatively regulates autophagy (Díaz-Troya et al., 2008). TOR activity is regulated by nutritional signals and environmental stresses (Dobrenel et al., 2016; Fu et al., 2020). Under normal conditions, *Arabidopsis* TOR is active and maintains the hyperphosphorylated state of its substrate, ATG13, thereby inhibiting the binding of ATG13 to ATG1 (Suttangkakul et al., 2011). Under nutrient-deficient conditions, TOR becomes inactivated, resulting in the dephosphorylation of ATG13, which triggers interaction between ATG13 and ATG1. ATG1-ATG13 then interact with two other subunits, ATG11 and ATG101, to form a complex which mediates autophagy initiation. The ATG1-ATG13-ATG11-ATG101 complex activates the subsequent steps of autophagy, including vesicle nucleation and phagophore expansion and closure (Suttangkakul et al., 2011; Li and Vierstra, 2014). The ATG9-ATG2-ATG18 complex is responsible for autophagosome formation and phagophore expansion via delivery of additional lipids (Yamamoto et al., 2012; Zhuang et al., 2017; Kang et al., 2018). Simultaneously, ATG8-phosphatidylethanolamine (PE) is inserted into the membrane of the expanding phagophore via a ubiquitin-like conjugation pathway (Ohsumi, 2001; Minina et al., 2018). Immature ATG8 precursors are transformed to mature ATG8 by the cysteine protease ATG4, and then are activated by ATP-dependent ubiquitin-activating enzyme (E1)-like ATG7 (Yoshimoto et al., 2004; Woo et al., 2014; Minina et al., 2018). ATG8 is subsequently delivered to the ubiquitin-conjugating enzyme (E2)-like ATG3 and finally conjugated to PE by a complex of ubiquitin-ligase enzymes (E3)-like ATG12-ATG5-ATG16 (Chung et al., 2010). ATG8 is regarded as a marker of the autophagosome and plays a vital role in the specific recognition of autophagy cargoes through the interaction of ATG8 with autophagy receptors (Liu and Bassham, 2012). The outer membrane of the autophagosome finally fuses with the vacuolar membrane and releases the cargoes into the vacuolar lumen for degradation (Liu and Bassham, 2012).

## Activation of Autophagy Through the Tor Signaling Pathway

TOR belongs to the phosphatidylinositol 3 kinase (PI3K)-related kinase family and can form two distinct complexes, TORC1 and TORC2 (TOR complex 1 and 2) in yeast and mammals. Among these, TORC1 regulates protein synthesis and the initiation of autophagy (Díaz-Troya et al., 2008). The TORC1 complex also exists in plants and algae (Mahfouz et al., 2006; Shemi et al., 2015). In plants, TORC1 is consist of a catalytic subunit TOR, a regulatory subunit LST8 (lethal with sec thirteen 8), and RAPTOR (regulatory-associated protein of TOR). The *Arabidopsis* RAPTOR family, consisting of RAPTOR1A and RAPTOR1B, functions as the target recognition cofactor of TOR (Anderson et al., 2005; Deprost et al., 2005). It is demonstrated that RAPTOR1B is involved in TOR-mediated autophagy repression (Pu et al., 2017). Two putative homologs of LST8, LST8-1 and LST8-2, have been identified in *Arabidopsis* (Moreau et al., 2012), and LST8-1 participates in adaptation to long days and hormone signaling (Moreau et al., 2012; Kravchenko et al., 2015). In plants, the TOR complex regulates the initiation of autophagy by modulating the ATG1-ATG13 complex in response to stresses (Mugume et al., 2020). There are four ATG1 isoforms and two ATG13 isoforms encoded in the *Arabidopsis* genome (Huang et al., 2019b). Both ATG1 and ATG13 are reversibly phosphorylated by TOR (Van Leene et al., 2019). ATG13 contains a canonical TOS (TOR signaling) motif, which mediates the interaction of ATG13 with RAPTOR (Son et al., 2018). During nutrient starvation, ATG1a is hyperphosphorylated by the SnRK1 (sucrose non-fermentation1-related protein kinase 1) complex and ATG13a is dephosphorylated (Suttangkakul et al., 2011; Chen et al., 2017; Soto-Burgos and Bassham, 2017). The hyperphosphorylated ATG1 interacts with dephosphorylated ATG13 to form an active complex along with ATG11 and ATG101 to initiate autophagy (Suttangkakul et al., 2011; Li and Vierstra, 2014).

## Regulation of Autophagy by Nutrient Signals

Under nutrient starvation, one of the most effective ways for plants to adapt is to degrade excess cytoplasmic material through autophagy to allow nutrients to be reused (Masclaux-Daubresse et al., 2014). *Arabidopsis atg* (*atg5*, *atg7*, and *atg18*) mutants show more severe chlorosis and reduced survival rates compared with wild type under nitrogen deprivation conditions (Thompson et al., 2005; Xiong et al., 2005). N remobilization from the leaves into the seeds has been evaluated using the isotopic tracer method, wherein plants are fed with ^15^NO_3_ and subsequently ^15^N is detected in the seeds (Guiboileau et al., 2012). Under N-limited conditions, ^15^N remobilization is sharply decreased in *atg5*, *atg9*, and *atg18a* mutants compared with wild type (Guiboileau et al., 2012). Salicylic acid (SA) is accumulated in *atg* mutants and is responsible for the early leaf senescence phenotype of *atg* mutants. However, the ^15^N-remobilization defect was not related to the early leaf senescence phenotype of the *atg* mutants (Guiboileau et al., 2013). Correspondingly, the rosette leaves of the *atg* mutants contained more ammonium, amino acids, and proteins but less sugars and starch compared to wild type, indicating that autophagy may affect the C/N balance of plants (Guiboileau et al., 2013; Izumi et al., 2013). The free amino acid content in *atg* mutants is increased under nitrogen starvation, consistent with their observed nitrogen remobilization defect (Guiboileau et al., 2013; Masclaux-Daubresse et al., 2014).

Autophagy also plays an important role under short day conditions. In *Arabidopsis*, compared with the starchless mutant *pgm* (*phosphoglucomutase*), the double mutant *pgm atg5* displays early cell death in leaves under short day conditions and reduced production of free amino acids during the night, suggesting that autophagy regulates the production of free amino acids which can act as an energy source during carbon starvation (Izumi et al., 2013). In addition, *Arabidopsis atg*5 and *atg7* etiolated seedlings exhibit shorter hypocotyls and produce fewer free amino acids compared with wild type seedlings when grow on fixed-carbon medium, indicating that autophagy has wide influences on metabolism during carbon starvation (Avin-Wittenberg et al., 2015). During prolonged carbon starvation, but not under nitrogen deprivation, *Arabidopsis* ATG6 can be phosphorylated by SnRK1 to initiate autophagy via an ATG1-independent autophagy initiation pathway, indicating that the initiation of autophagy may be regulated by different mechanisms in response to nitrogen and carbon starvation (Huang et al., 2019b).

After sulfur is absorbed by plants in the form of sulfate, it is successively converted into sulfide by various enzymes, including ATP sulfurylase (ATPS), APS reductase (APR), and sulfite reductase (SIR) (Takahashi et al., 2011). The mutant *sir1-1* displays inhibited TOR activity and reduced glucose levels when grown in sulfate deprivation conditions (Dong et al., 2017). TOR activity in *sir1-1* can be restored by exogenous glucose, indicating that sulfur may regulate TOR activity through glucose signaling (Dong et al., 2017). In *Arabidopsis*, glucose may activate TOR by inhibiting the activity of SnRK1 (Baena-González and Hanson, 2017). SnRK1 directly phosphorylates RAPTOR1B, thus inhibiting TOR activity (Nukarinen et al., 2016). Alternatively, sulfur starvation may directly promote autophagy through other unknown pathways.

In *Arabidopsis*, D-glucose levels are sensed by heterotrimeric G protein complex and RGS1 (regulator of G-protein signaling 1), a member of a glucose-sensing complex (Chen and Jones, 2004). Turnover of the RGS1 protein is crucial for cell cycle regulation and the cellular glucose signaling pathway (Jiao et al., 2019). In *Arabidopsis*, the expressions of *ATGs* are much more induced in wild type than in *rgs1* when treated with 1% glucose, indicating that RGS1 promotes autophagy (Yan et al., 2017). Furthermore, it was demonstrated that proteasome-independent degradation of RGS1, as well as autophagic flux, can be induced by nutrient starvation. RGS1 directly interacts with ATG8 and co-localizes with autophagosomes in *Arabidopsis* root cells, indicating that autophagy mediates the degradation of RGS1 in response to sugar signaling (Jiao et al., 2019).

It has been reported that ER stress-dependent autophagy can be rapidly activated in plant roots in response to phosphate (Pi) starvation (Naumann et al., 2019). *Arabidopsis LPR1* (*LOW PHOSPHATE RESPONSE 1*) encodes a cell wall-targeted ferroxidase (Müller et al., 2015), which is involved in deposition of callose and pectin polymers in cell walls, inhibition of cell-to-cell communication and maintenance of the root apical meristem under Pi deficiency conditions (Hoehenwarter et al., 2016). LPR1 activity can be inhibited by PDR2 (PHOSPHATE DEFICIENCY RESPONSE2), the ER-resident AtP5A, which is involved in the ER stress response (Ticconi et al., 2009; Müller et al., 2015; Sørensen et al., 2015). Thus, local root Pi sensing and ER stress-dependent autophagy initiation are connected by the PDR2-LPR1 module (Naumann et al., 2019). It has been reported that the S-domain receptor kinase (ARK2) and U box/Armadillo repeat-containing E3 ligase (PUB9) regulate lateral root growth under Pi deficiency conditions in *Arabidopsis* (Sankaranarayanan and Samuel, 2015). During Pi starvation, activation of ARK2 results in phosphorylation of PUB9, which is likely involved in the selective degradation of AUX/IAA (Auxin/Indole-3-Acetic Acid) protein or other repressors of auxin accumulation via autophagy. This leads to an increase of auxin in lateral root initiation sites (Sankaranarayanan and Samuel, 2015).

**FIGURE 2:**
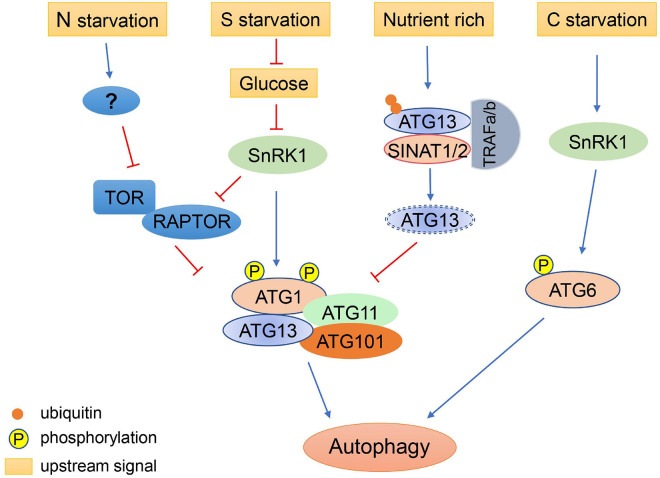
Regulation of autophagy initiation under different nutritional conditions. Autophagy is induced by nitrogen (N) starvation in plants through inhibition of TOR. Sulfur (S) starvation regulates autophagy through the repression of glucose signaling, resulting in the activation of SnRK1, which inhibits TOR and activates the ATG1 autophagy initiation complex. Under nutrient-rich conditions, ATG13 is ubiquitinated by the SINATs-TRAF1s complex and degraded via the proteasome pathway, maintaining low levels of autophagy. Under prolonged carbon (C) starvation, ATG6 can be phosphorylated by SnRK1 to initiate autophagy via an ATG1-independent pathway.

Over-induction of autophagy can impair the normal functions of plant cells, particularly during prolonged starvation or during nutrient recovery after starvation. Therefore, it is sometimes necessary to inhibit autophagy to ensure that it is maintained at a reasonable level. It was reported that *Arabidopsis* RING-type E3 ligases SINAT1 (SEVEN *IN ABSENTIA* OF ARABIDOPSIS THALIANA1), SINAT2, and SINAT6 regulate ATG1 and ATG13 levels via ubiquitination of ATG13, thereby regulating the dynamics of autophagy (Qi et al., 2019). During nutrient-rich conditions, ATG13 is ubiquitinated by the SINAT1-SINAT2-TRAF1a-TRAF1b (TUMOR NECROSIS FACTOR RECEPTOR ASSOCIATED FACTOR) complex and degraded via the ubiquitin proteasome pathway, thus maintaining low levels of autophagy (Qi et al., 2019). Under prolonged nutrient starvation, ATG13 is similarly degraded through the ubiquitin proteasome pathway, mediated by SINAT1 and SINAT2, to downregulate autophagy (Qi et al., 2019). In contrast, SINAT6 has an opposite effect, whereby it competitively interacts with ATG13 to inhibit the ubiquitination and degradation of the ATG1/ATG13 complex, leading to activation of autophagy during nutrient starvation (Qi et al., 2019). These results suggest that regulation of autophagy by various intracellular signals is a very delicate process, which ensures that plants can respond to environmental changes in an appropriate and timely manner (Figure 2).

## Regulation of Autophagy by Biotic Stress Signals

Plants often suffer from infection by pathogens or plant viruses, which can stimulate the host innate immunity of plants. As the signal source, the pathogen can be recognized by membrane-localized or cytoplasmically-localized receptors, thereby triggering an immune response (Peng et al., 2018). Autophagy is induced by many pathogens’ recognition receptors to directly target intracellular pathogens for selective degradation (Deretic, 2012). In *Nicotiana benthamiana*, *Rice stripe virus* (RSV) infection is promoted in *atg5* or *atg7* mutants. It has been shown that NbP3IP can interact with p3, an RNA silencing suppressor protein encoded by RSV, to mediate the autophagic degradation of p3 and suppression of the RSV infection (Jiang et al., 2021a). NbP3IP is a potential selective autophagy cargo receptor and can interact with NbATG8f. Similarly, the rice homolog OsP3IP also mediates p3 degradation via interaction with OsATG8b and p3 (Jiang et al., 2021a). When CFP-NbATG8f was co-expressed with NbP3IP-Myc in *Nicotiana benthamiana*, an increase in CFP -labeled autophagic structures was detected, indicating that P3IP participates in the regulation of autophagy during the plant viral defense response (Jiang et al., 2021b). In *Arabidopsis*, a small 71 amino acid peptide, VISP1, can be induced by *Cucumber mosaic virus* (CMV) infection and interacts with ATG8 through UIM (ubiquitin-interacting motif) motif. VISP1 functions as a selective autophagy receptor involved in degradation of SGS3 (suppressor of gene silencing 3)/RDR6 (RNA-dependent RNA polymerase 6)-bodies to negatively regulate siRNA amplification, resulting in suppression of antiviral immunity (Tong et al., 2021).

Autophagy can also prevent the spread of pathogen infection by promoting hypersensitive response programmed cell death (HR-PCD) (Zhou et al., 2014). Autophagy-deficient *Arabidopsis atg7* and *atg9* mutants show a weakened PCD response after infection with *Pseudomonas syringae* pv. tomato DC3000 harboring the avirulence gene *AvrRPS4* (Hofius et al., 2009). Thus, in this case, autophagy limits the spread of the pathogen by promoting cell death. But on the other hand, during the immune response of plants to avirulent biotrophic pathogens and necrotrophic pathogens, autophagy can restrict the spread of unnecessary cell death to promote cell survival (Teh and Hofius, 2014; Zhou et al., 2014). HR-PCD is induced at the site of infection when wild type *Nicotiana benthamiana* leaves are infected with *Phytophthora infestans* or *Pseudomonas syringae* pv. tomato DC3000. In *Nicotiana benthamiana* autophagy-deficient plants, HR-PCD spreads uncontrollably to uninfected cells (Liu et al., 2005). In *Arabidopsis*, *Psm* ES4326/*AvrRpt2* can trigger autophagy-dependent HR via the EDS1 (enhanced disease susceptibility 1) pathway, but NPR1 (non-expressor of pathogenesis-related genes 1) suppresses the autophagy flux (Gong et al., 2020). In *Arabidopsis atg4a4b* double mutant, total SA (salicylic acid) content decreased and free SA unchanged compared with wild type after *AvrRpt2* infection, indicating that ATG4 can suppress the consumption of free SA. ATG4 inhibits SA consumption resulting in the inhibition of NPR3 (non-expressor of pathogenesis-related genes 3) synthesis and enhanced expression of NPR1, which inhibits autophagy durin*g AvrRpt2* affection. These results indicate that ATG4 mediates *AvrRpt2*-induced autophagy dependent on SA (Gong et al., 2020). Therefore, the role of autophagy in the immune response depends on what pathogen signals the plant cell receives.

**FIGURE 3:**
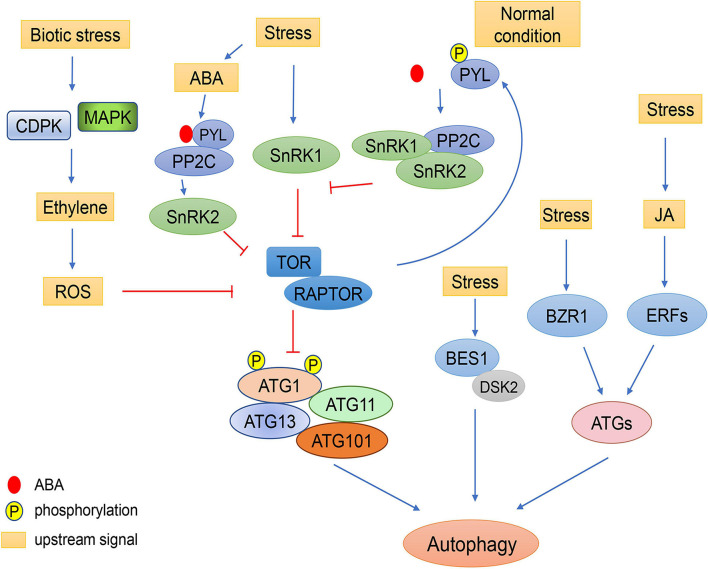
Schematic diagram of autophagy regulation by environmental stress through plant hormones. Under stress conditions, the concentration of ABA increases. ABA receptors, PYLs, bind to ABA and PP2C, resulting in the activation of SnRK2. SnRK2 phosphorylates RAPTOR and inactivates the TOR complex to induce autophagy. Under normal conditions, TOR kinase phosphorylates the PYL ABA receptors, preventing PYL from binding to ABA and PP2C. SnRK2 and PP2C form repressor complexes which interact with SnRK1 and prevent it from interacting with TOR. In response to biotic stress, ethylene signaling is activated by MAPK and CDPK. The ethylene signaling pathway may regulate autophagy through ROS signaling. Abiotic stresses also regulate autophagy through the JA pathway, in which the expression of *ATGs* can be induced by ERFs. Additionally, cold can enhance the stability of BZR1 to increase expression of *ATGs*, resulting in enhanced autophagy. The brassinosteroid-responsive transcription factor BES1 is selectively degraded by DSK2-mediated autophagy under starvation and drought stresses.

Furthermore, some pathogens can even facilitate their infection in plants by regulating autophagy. The γb protein from *Barley stripe mosaic virus* can directly interacts with *Nicotiana benthamiana* ATG7 to interfere with the interaction of ATG7 with ATG8f in a competitive manner, resulting in the suppression of autophagy (Yang et al., 2018). In *Arabidopsis*, TOR activity is inhibited by the effector AWR5 (alanine-tryptophan-arginine tryad) secreted from the bacterium *Ralstonia solanacearum* to help feed the pathogen through autophagy induction (Popa et al., 2016). Additionally, autophagy can be induced by activation of salicylic acid signaling in a NPR1-dependent manner when *Arabidopsis* is infected with *P. syringae* (Yoshimoto et al., 2009). Moreover, a type III effector (T3E), Hrp outer protein M1 (HopM1), secreted by *P. syringae* can interact with proteasomes to promote the selective degradation of proteasomes via autophagy, inhibiting salicylic acid-dependent defense responses (Üstün et al., 2018). However, NBR1 (NEIGHBOR OF BRCA1)-dependent selective autophagy prevents the formation of HopM1-mediated water-soaked lesions and thereby dampens bacterial virulence and proliferation (Üstün et al., 2018). In summary, the roles of autophagy in the plant immune system are the result of interactions between various signaling pathways (Figure 3).

## Induction of Autophagy by Abiotic Stress Signals

Autophagy can be induced by various abiotic stresses, including heat, cold, drought, osmotic, and salt stress, to improve plant resistance (Slavikova et al., 2008; Dündar et al., 2019; Sedaghatmehr et al., 2019; Bao et al., 2020; Chi et al., 2020; Jung et al., 2020; Thirumalaikumar et al., 2021). TOR kinase, which is crucial for autophagy induction, works downstream of SnRK1 (Soto-Burgos and Bassham, 2017). In response to abiotic stress, *Arabidopsis* trehalose 6-phosphate (T6P) suppresses SnRK1 activity, which may result in inhibition of autophagy (Tsai and Gazzarrini, 2014; Soto-Burgos and Bassham, 2017). SnRK1 inhibits the activity of TOR by phosphorylating RAPTOR1B. In *Arabidopsis*, KIN10, a SnRK1 catalytic subunit, can also phosphorylate RAPTOR1B (Nukarinen et al., 2016). Overexpression of *KIN10* increases autophagy, whereas knockout of *KIN10* inhibits abiotic stress-induced autophagy in *Arabidopsis*. SnRK1 can also regulate autophagy in a TOR-independent manner, during which the ATG1 complex can be phosphorylated and activated directly by SnRK1 to induce autophagy (Chen et al., 2017; Soto-Burgos and Bassham, 2017).

The protein phosphatase 2A-associated protein (PP2A-associated protein, TAP46), a regulatory subunit of PP2A, can be phosphorylated by TOR kinase, inducing PP2A activity to inhibit the formation of the double-membraned autophagosomes (Máthé et al., 2021). Silencing of *TAP46* activates autophagy in tobacco and *Arabidopsis*, and the *tap46* mutant shows the same phenotype as *tor* mutants, including TOR inactivation resulting in induction activation of autophagy and nitrogen mobilization, indicating that TAP46 negatively regulates autophagy via TOR signaling (Ahn et al., 2011).

Plants can also regulate autophagy independently of TOR kinases. Under carbon starvation, *Arabidopsis* KIN10 phosphorylates ATG6 to activate the PI3K complex and initiate autophagy (Huang et al., 2019b). Under oxidative stress and ER stress, KIN10 can induce autophagy by directly activating the ATG1 complex (Chen et al., 2017). Levels of phosphorylated ATG1 are increased by overexpression of KIN1, and KIN10 can interact with ATG1a and ATG13a *in vitro*, indicating that KIN10 most likely phosphorylates ATG1 in TOR-independent autophagy (Chen et al., 2017; Figure 3).

ATG8, a marker of the autophagosome, is involved in the specific recognition of autophagy cargoes. In plants, ATG8 also plays an important role in the regulation of autophagy in response to abiotic stresses. In *Arabidopsis*, a plant-specific developmental regulatory protein COST1 (constitutively stressed 1) directly interacts with ATG8e to inhibit autophagy under normal conditions to promote plant growth. Under drought stress, COST1 is degraded via the ubiquitin proteasome system (UPS) and autophagy, releasing ATG8 to promote autophagosome formation and drought tolerance (Bao et al., 2020).

The expression of multi-stress regulatory genes which encode plant tryptophan-rich sensory protein (TSPO) can be induced by osmotic stress and salt stress (Guillaumot et al., 2009). AtTSPO directly binds to ATG8 through the AIM domain of AtTSPO and also interacts with a plasma membrane aquaporin, PIP2;7, to mediate the autophagic degradation of PIP2;7, which subsequently reduces intercellular water transport under water deficient conditions (Vanhee et al., 2011; Hachez et al., 2014). The degradation of PIP2;7 can be triggered by abscisic acid (ABA) treatment, indicating that ABA may participate in selective autophagy in response to osmotic stress (Hachez et al., 2014). Two plant specific proteins, ATI1 and its homolog ATI2, can also interact with ATG8 via putative AIM motifs during salt stress to mediate the degradation of chloroplast membranes and fragments via autophagy, indicating that the selective degradation mediated by ATI1 and ATI2 is involved in salt tolerance in plants (Honig et al., 2012; Michaeli et al., 2014). In *Arabidopsis*, ATI1 and ATI2 are also involved in the regulation of reticulophagy induced by starvation stress during darkness (Wu et al., 2021). Under carbon starvation, ATI1 and ATI2 interact with ER-localized MSBP1 (Membrane Steroid Binding Protein 1) to mediate the autophagic degradation of MSBP1, indicating that ATI1 and ATI2 serve as MSBP1 cargo receptors to regulate the selective reticulophagy during starvation stress (Wu et al., 2021).

## The Interaction Between Hormone Signaling and Autophagy

Phytohormones play comprehensive and vital roles during plant growth and development under normal and adverse conditions (Gou et al., 2019). Recently, considerable progress has been made to uncover the crosstalk between autophagy and plant hormone signals (Figure 3).

ABA is an essential endogenous signal which acts during various stresses in plants to regulate leaf expansion and stomatal closure (Raghavendra et al., 2010). Under osmotic stresses such as drought and high salinity, ABA accumulates in all plant organs, indicating ABA is a systemic signaling molecule (Yoshida et al., 2014). Under stress conditions, TOR activity can be inhibited by ABA signaling to induce autophagy (Wang et al., 2018b). SnRK2, as a kinase downstream of ABA, can phosphorylate RAPTOR and inactivate the TOR complex (Wang et al., 2018b). But under normal conditions, TOR kinase phosphorylates PYL (pyrabactin resistance1/PYR1-like) ABA receptors, preventing the PYL receptors from binding to ABA and PP2C phosphatase effectors, resulting in the inactivation of SnRK2 kinases (Wang et al., 2018b). In the absence of ABA, SnRK2 and PP2C are involved in the formation of a repressor complex which interacts with SnRK1 and prevents it from interacting with TOR. However, in the presence of ABA, this repressor complex is disassembled and SnRK1 is released to inhibit the activity of TOR (Belda-Palazón et al., 2020). Moreover, there are a number of ABA responsive elements in the promoters of *ATGs* (Yue et al., 2018), suggesting that the ABA signaling pathway and autophagy can regulate each other alternatively according to environmental conditions to ensure the normal growth of plants.

Ethylene is another stress response phytohormone. It was shown that the ethylene signaling pathway increases ROS levels. It was shown that antioxidant systems can also be activated by ethylene to protect cells from excessive ROS damage (Mersmann et al., 2010; Jiang et al., 2013; Yamauchi et al., 2014). The production of ROS by stress can cause damage to proteins and organelles. These damaged cellular components will then need to be cleared by autophagy (Signorelli et al., 2019). However, the relationship between ethylene signaling and autophagy is still elusive. In the autophagy-deficient mutants *atg5* and *atg9*, a series of ethylene signaling genes are induced (Masclaux-Daubresse et al., 2014). Under drought stress, the transcription factor ERF5 (Ethylene Response Factor 5) binds to the promoters of *ATG8* and *ATG18* to increase their expression and autophagy (Masclaux-Daubresse et al., 2014). More work is still needed to uncover the specific mechanisms of how ethylene signaling affects autophagy.

Crosstalk between brassinosteroid (BR) signaling and autophagy has recently been revealed. In *Arabidopsis*, TOR signaling can regulate the protein level of the transcription factor BZR1 (brassinazole-resistant 1), a key regulatory factor in BR signaling, to maintain plant growth and development under carbon starvation. Decreased expression of TOR results in reduced expression of BR-responsive genes and subsequently inhibition of plant growth. This phenotype can be partially recovered by the increase of active forms of BZR1 (Zhang et al., 2016). In addition, *Arabidopsis* ribosomal protein S6 kinase 2 (S6K2), a direct downstream target of TOR, can directly phosphorylate the BR signaling component BIN2 (brassinosteroid insensitive 2), indicating the TOR-S6K2-BIN2 signaling pathway mediates plant growth (Xiong et al., 2017). Another BR signaling positive regulator, transcription factor BRI-EMS SUPPERSSOR 1 (BES1), is selectively degraded through a DOMINANT SUPPRESSOR OF KAR 2 (DSK2)-mediated autophagy pathway under starvation and drought stresses in *Arabidopsis* (Nolan et al., 2017). BR signaling also regulates autophagy in turn. Under brassinolide treatment, the expression of *ATGs* and the formation of autophagosomes are reduced in *BZR1-silenced* tomato plants, but are enhanced in *BZR1* overexpressing tomato plants, suggesting feedback regulation of BR signaling on autophagy (Wang et al., 2019). Recently, it has been found that cold and BR can enhance the stability of BZR1 that can bind to the promoter of *ATGs* and *NBR1* to increase the expression of these genes resulting in enhanced autophagy in tomato. The increased autophagy promotes the degradation of damaged insoluble ubiquitinated proteins though selective autophagy receptor NBR1-mediated pathway, indicating that BR positively regulates NBR1-dependent selective autophagy in response to cold (Chi et al., 2020).

Auxin was discovered in maize and in *Arabidopsis* suspension culture to activate TOR kinase, as evidenced by increased phosphorylation of T449 on S6K1, a direct downstream target of TOR (Beltrán-Peña et al., 2002; Turck et al., 2004; Schepetilnikov et al., 2013). Auxin biosynthesis induced by light and glucose synthesized by photosynthesis activate TOR and promote the growth of shoot meristems in *Arabidopsis* (Cai et al., 2017; Li et al., 2017). In *Arabidopsis*, ROP (Rho-like GTPases from plants) small GTPases member, ROP2, directly activates TOR kinase in response to auxin (Li et al., 2017; Schepetilnikov et al., 2017). Furthermore, an E3 ubiquitin ligase COP1 (Constitutive Photomorphogenesis 1) functions as an upstream regulator of ROP2 and negatively regulates auxin-ROP2-TOR signaling pathway in response to light (Cai et al., 2017). Agreeing with auxin’s ability to activate TOR, adding exogenous auxin to seedlings under various abiotic stress conditions can inhibit autophagy (Pu et al., 2017). In response to glucose stress, *Arabidopsis* sugar sensor HXK1 (hexokinase 1) and energy sensor KIN10 suppress TOR activity to inhibit constitutive autophagy modulating function of the peroxisomes, which contributes to the production of ROS and IAA to regulate root meristem (Huang et al., 2019a). Induction of auxin signaling can also activate the differentiation of xylem cells. Autophagy is also induced during tracheary element differentiation, although the underlying mechanism is not clear (Ibañes et al., 2009; Kwon et al., 2010). As an activator of TOR, an increase in auxin leads to induction of TOR activity, which occurs simultaneously with the induction of autophagy in the xylem, suggesting that autophagy is activated despite the presence of active TOR (Pu et al., 2017). The BR signaling is required for proper arrangement of the plant-shoot vasculature (Ibañes et al., 2009). In response to BR, autophagy is activated through the activation of RabG3b, a small GTP binding protein, to promote the differentiation of tracheary elements (Kwon et al., 2010). These observations imply that autophagy may be regulated through additional unknown pathways other than the TOR signaling in the xylem.

The effect of phytohormone jasmonic acid (JA) signaling on downstream genes can be affected by TOR inhibition (Dong et al., 2015). JA has been shown to regulate the expression of *ERFs*, which encode an inducer of resistance to abiotic stresses, and JA-related ERF elements are widely present in the promoters of *ATGs*, indicating crosstalk between JA signaling and autophagy (Yue et al., 2018; Figure 3). JA is also involved in the response to biotic stress. In *Arabidopsis*, WRKY33 not only regulates the expression of resistance genes in response to fungal pathogens through JA signaling, but can also interact with ATG18a to regulate autophagy (Lai et al., 2011). High levels of the phytohormone SA can lead to autophagy-related PCD in *Arabidopsis*, and the induction of PCD during senescence in *atg5* is via SA signaling (Yoshimoto et al., 2009). Metabolomic and transcriptomic analyses have shown that SA biosynthesis is up-regulated in *Arabidopsis atg5* mutant under low nitrate conditions (Masclaux-Daubresse et al., 2014). It was proposed that a ROS burst is induced by SA accumulation, which in turn promotes autophagy to reduce ROS, indicating negative feedback (Masclaux-Daubresse et al., 2014). However, the regulatory mechanisms connecting SA signaling and autophagy are still unclear.

## Future Perspectives

The development of a plant from a seed into a mature organism involves the continuous differentiation of new organs and the degeneration of old organs. Autophagy is not only responsible for the removal of cellular waste components, but also plays key regulatory roles in different developmental stages and in response to a wide range of biotic and abiotic environmental stresses. However, excessive autophagy can also damage normal structural components of cells. The level of autophagy is carefully regulated by plants through a variety of pathways according to changing environmental conditions. Environmental signals regulate the initiation of autophagy by affecting TOR kinase activity or though other TOR-independent pathway. Many plant hormones also regulate autophagy, which in turn also provides feedback regulation on these signaling pathways, which jointly ensures the normal growth and development of plants. Recent studies have also deciphered the regulation of autophagosome biogenesis. For example, under ER stress, the phospholipid-binding activity of ATG18a can be activated by reversible persulfidation at Cys103 to negatively regulate autophagosome formation and ensure the appropriate physiological response (Aroca et al., 2021). During nutrient starvation, coat protein complex II (COPII) vesicles act as a membrane source for autophagosome formation, mediated by the specific recognition of ATG8e by a COPII component, AtSar1d (Zeng et al., 2021). ROP8, a member of the Rho family guanosine triphosphatases (GTPases) of plants (ROPs), promotes autophagosome formation via its downstream effector Sec5, indicating a previously unappreciated role of ROP8-Sec5 signaling in autophagy promotion (Lin et al., 2021).

Despite the recent achievements uncovering the interactions between signaling pathways and autophagy in plants, there are still many open questions. As an important regulator of autophagy, how does TOR accurately identify different external signals to make the appropriate responses? How are stress signals, ROS signals, and plant hormone signals, which can all regulate autophagy levels, related?How do plants accurately regulate the dynamics of autophagy? The integrated regulatory mechanisms connecting stresses, plant hormones, and autophagy in plants are still unclear. The study of the molecular mechanisms by which environmental signals regulate autophagy is crucial to uncover these mysteries.

## Author Contributions

PW, TW, TS, and CM conceived and wrote the review. JH, ML, and YZ contributed to revision of the manuscript. All authors have read and agreed to the published version of the manuscript.

## Conflict of Interest

The authors declare that the research was conducted in the absence of any commercial or financial relationships that could be construed as a potential conflict of interest.

## Publisher’s Note

All claims expressed in this article are solely those of the authors and do not necessarily represent those of their affiliated organizations, or those of the publisher, the editors and the reviewers. Any product that may be evaluated in this article, or claim that may be made by its manufacturer, is not guaranteed or endorsed by the publisher.
